# Supplementary material for: Prevalence and determinants of work-related ocular symptoms among dentists of Bangladesh: A cross-sectional study
Source: PLoS One. 2025 Dec 11;20(12):e0336159. doi: 10.1371/journal.pone.0336159 (PMC12697935; doi:10.1371/journal.pone.0336159)
Supplement: S3 File — (DOCX) [file pone.0336159.s003.docx]

**APPENDIX-A**

**Informed Consent Form**

**NORTH SOUTH UNIVERSITY**

ID: ………………………………………… Date: ………………………….

Name of the Respondent:

……………………………………………………………………………………

We, a group of young researchers, are conducting research on **“Prevalence and Determinants of Work-related Ocular Symptoms Among Dentists of Bangladesh: A Cross-sectional Study”** Dr. Md. Delwer Hossain Hawlader, Associate Professor & Chairman, North South University, is supervising this study. If you have any questions about this study, please contact Dr. Md. Delwer Hossain Hawlader at [mohammad.hawlader@northsouth.edu](mailto:mohammad.hawlader@northsouth.edu).

As a part of this study, your participation would be highly appreciated and would contribute a lot to this research study. You will be asked to answer several questions. Your identity will not be disclosed and will be kept confidential.

Your participation in this study will not involve any inconvenience or risks. If any question asked to you during the study poses embarrassment or discomfort, you are free to refuse to answer those questions. Your participation is voluntary. Refusal to participate or withdrawal of your consent or discontinuing participation in the study will not result in any penalty or loss of benefits. The results of this study will be presented anonymously.

North South University has reviewed and approved the procedures of this study. If you have any questions about this study, you should feel free to ask now or anytime throughout the study. If you have understood the nature of the study and have agreed to participate, please sign in the place, indicated below.

| …………………….….…..…………….. | ……….….…..………………………….. |
| --- | --- |
| Investigator’s Signature &Date | Participant’s Signature &Date |

**APPENDIX-B QUESTIONNAIRE**

**Title: Prevalence and Determinants of Work-related Ocular Symptoms Among Dentists of Bangladesh: A Cross-sectional Study**

| **Identification No __________________** | | |
| --- | --- | --- |
| **Socio-demographic information** | | |
| - 1. Age (in years): _____________________________ | | |
| - 1. Gender | - Male - Female | |
| - 1. Marital status | - Married - Unmarried | |
| **Life style related variables:** | | |
| - 1. Personal Habit: | | - Smoking - Tea/Coffee - Betel quit - Nothing - Others |
| - 1. The habit of doing physical exercise: | | - Never - Occasional - Regular |
| - 1. How will you define your sleep? | | - Sound sleep - Average - Poor |
| - 1. How will you define your diet? | | - Nutritious - Non nutritious - Occasional non nutritious |

| **Work related factors** | |
| --- | --- |
| 8.Type of job | - Public organization - Private organization - Self employed - Not currently practicing dentistry |
| 9. Number of Practicing Day per week- (How many days write in number) | |
| 10. Practicing hours per day- (How many hours write in number) | |
| 11. Duration of dentistry practice in years- (How many months write in number) | |
| 12. The average number of patients attended in a day- (Write in number) | |
| 13. Break time after one treatment session | - No break times - 5 to 10 minutes - 10 to 20 minutes - More than 20 minutes |
| 14.Contact with amalgam- | - No contact - Irregular - Regular |
| 15.Use of Personal Protective Equipment in daily practice: | - Musk - Surgical gloves - Plastic Apron or Gowns Head Covering - Protective Eye wear - others: |
| 16.The practice of using eye protector- | - Never - Irregular - Regular |
| 17.Use of Light-curing units by - | - avoiding looking directly at the light probe - looking at it from a safe distance (min 25 cm) |
| 18.Use of Lasers- | - Never - Irregular - Regular |
| 19. Facility of cooling system in  workplace: | - Yes - No |
| 20. Model of chair used during dental practice: | |

| **Clinical factors** | |
| --- | --- |
| 21.Height feet and Inch - |  |
| 22.Weight (in KG) - |  |
| 23.Do you use Lense or Spectacles? | - Lense - Spectacles - Both Lense and spectacles - Nothing |
| 24.If you use Lense or Spectacles, please mention the duration of use of it | |
| 25.H/O eye injury - | - Never - Yes (During dental practice) - Yes (Other than dental practice) |
| 26.Reason of last eye care visit - | - Routine check - Difficulty in reading - Redness of eye - Pain - Itching - Double vision - Others |
| 27.H/O any existing Diseases- | - Headache or Migraine - Short-sightedness - Long-sightedness - Astigmatism - Cataract - Glaucoma - Repeated Conjunctivitis - Diabetes mellitus - Hypertension - Others: |
| 28.Drug History (Regular intake of any drug) |  |

| **Questionnaire regarding Eye Problems** | | | |
| --- | --- | --- | --- |
| **29. Problems in eye (Frequency)** | **Never** | **Occasionally** | **Often or always** |
| Burning |  |  |  |
| Itching |  |  |  |
| Feeling of a foreign body |  |  |  |
| Tearing |  |  |  |
| Excessive blinking |  |  |  |
| Redness |  |  |  |
| Pain |  |  |  |
| Heavy eyelid |  |  |  |
| Dryness |  |  |  |
| Blurred vision |  |  |  |
| Double vision |  |  |  |
| Difficulty focusing for near vision |  |  |  |
| Increased sensitivity to light |  |  |  |
| Colored halos around objects |  |  |  |
| Feeling that sight is worsening |  |  |  |

**APPENDIX-C CONSENT FORM (BENGALI)**

**সম্মতিপত্র**

**নর্থ সাউথ ইউনিভার্সিটি**

আইডি নং: তারিখ:

উত্তরদাতার নাম:

আমরা, নর্থ সাউথ বিশ্ববিদ্যালয়ের এমপিএইচ প্রোগ্রামের একদল তরুণ গবেষক, একটি গবেষণা কর্ম করছি যার শিরোনাম হল “**Prevalence and Determinants of Work-related Ocular Symptoms Among Dentists of Bangladesh: A Cross-sectional Study**”। নর্থ সাউথ ইউনিভার্সিটির সহযোগী অধ্যাপক ও চেয়ারম্যান ডাঃ মোঃ দেলোয়ার হোসেন হাওলাদার গবেষণাটি তত্ত্বাবধান করছেন। এই গবেষণা সম্পর্কে আপনার কোনো প্রশ্ন থাকলে, অনুগ্রহ করে ডাঃ মোঃ দেলোয়ার হোসেন হাওলাদার স্যার এর সাথে mohammad.hawlader@northsouth.edu-এই ঠিকানায় যোগাযোগ করুন। আপনাকে উক্ত গবেষণা কর্মে কিছু প্রশ্নের উত্তরও দিতে হবে যা এই ফর্মে উল্লেখ করা আছে।

আমরা আপনাকে জানাতে চাই যে এটি সম্পূর্ণরূপে একটি একাডেমিক গবেষণাকর্ম এবং আপনার প্রদত্ত তথ্য সমূহ অন্য কোন উদ্দেশে ব্যবহৃত হবে না। আপনার নাম প্রকাশনায় গোপন থাকবে।

এই গবেষণা কর্মে আপনার অংশগ্রহণ ঐচ্ছিক এবং গবেষণাকর্মের যেকোন সময় এতে অংশ নেয়া থেকে বিরত থাকতে পারবেন। ইন্টারভিউ চলাকালীন কোন নির্দিষ্ট প্রশ্নের উত্তর না দিতে চাইলে, প্রশ্নের উত্তর না দেয়ার অধিকার আপনি সংরক্ষণ করেন।

আমরা আপনার সহযোগিতায় কৃতজ্ঞ থাকব। আপনি যদি গবেষণায় যোগ দিতে সম্মত হন, তবে অনুগ্রহ পূর্বক নির্দিষ্ট স্থানে স্বাক্ষর করুন।

………………………………………………………. ………………………………………………………

তদন্তকারীর স্বাক্ষর এবং তারিখ অংশগ্রহণকারীর স্বাক্ষর এবং তারিখ

**APPENDIX-D**

**QUESTIONNAIRE (BENGALI)**

**Title:** **Prevalence and Determinants of Work-related Ocular Symptoms Among Dentists of Bangladesh: A Cross-sectional Study**

| **তথ্য সনাক্তকরণ তারিখ** | | | | | দিন | মাস | | সাল |
| --- | --- | --- | --- | --- | --- | --- | --- | --- |
| সামাজিক জনসংখ্যা সংক্রান্ত তথ্য | | | | | | | | |
| 1. বয়স (বছরে)- সংখ্যায় লিখবেন | | 1. লিঙ্গ | ☐ পুরুষ  ☐ মহিলা | | 1. বৈবাহিক অবস্থা | | ☐ বিবাহিত  ☐ অবিবাহিত | |
| **জীবনধারা বিষয়ক তথ্য** | | | | | | | | |
| 1. ব্যক্তিগত অভ্যাস | ☐ ধূমপান  ☐ চা/কফি  ☐ পান- সুপারি  ☐ কিছুই না | | 1. শারীরিক ব্যায়াম করার অভ্যাস | | | | ☐ কখনই না  ☐ মাঝে মাঝে  ☐ নিয়মিত | |
| 1. আপনি কিভাবে আপনার ঘুম সংজ্ঞায়িত করবেন? | ☐ ভালো ঘুম  ☐ গড়  ☐ যথেষ্ট নয় | | 1. আপনি কিভাবে আপনার খাদ্য সংজ্ঞায়িত করবেন? | | | | ☐ পুষ্টিকর  ☐ পুষ্টিহীন  ☐ মাঝে মাঝে পুষ্টিহীন | |
| **পেশা সম্পর্কিত তথ্য** | | | | | | | | |
| 1. কাজের ধরন | | ☐ পাবলিক প্রতিষ্ঠান  ☐ বেসরকারী প্রতিষ্ঠান  ☐ স্ব-নিযুক্ত  ☐ বর্তমানে ডেন্টাল প্র্যাক্টিস করছেন না | | | | | | |
| 1. প্রতি সপ্তাহে ডেন্টাল প্র্যাক্টিস দিনের সংখ্যা | সংখ্যায় লিখবেন | 1. প্রতিদিন কত ঘন্টা ডেন্টাল প্র্যাক্টিস করা হয়? | | | | | সংখ্যায় লিখবেন | |
| 1. বছরের মধ্যে ডেন্টাল প্র্যাক্টিসে সময়কাল | সংখ্যায় লিখবেন | 12. একদিনে রোগীর সংখ্যা গড়ে | | | | | সংখ্যায় লিখবেন | |
| 1. একটি চিকিত্সা সেশনের পরে বিরতি সময় | ☐ বিরতির সময় নেই  ☐ ৫- ১০ মিনিট  ☐ ১০ - 20 মিনিট  ☐ 20 মিনিটের বেশি | | | | | | | |
| 1. অ্যামালগামের সাথে সংস্পর্শ- | ☐ কোন সংস্পর্শ নেই  ☐ অনিয়মিত  ☐ নিয়মিত | | | | | | | |
| 1. দৈনন্দিন প্র্যাক্টিসে ব্যক্তিগত সুরক্ষামূলক সরঞ্জামের ব্যবহার | ☐ মাস্ক  ☐ সার্জিক্যাল গ্লাভস  ☐ প্লাস্টিকের এপ্রোন বা গাউন  ☐ হেড কাভারিং  ☐ প্রতিরক্ষামূলক আই ওয়ার  ☐ অন্যান্য | | | | | | | |
| 1. চোখের সুরক্ষাকারী ব্যবহার করার অভ্যাস- | ☐ কখনই না  ☐ অনিয়মিত  ☐ নিয়মিত | | | | | | | |
| 1. লাইট-কিউরিং ইউনিটের ব্যবহার- | ☐ লাইট প্রোবের দিকে সরাসরি তাকানো এড়িয়ে যাওয়া  ☐ এটিকে নিরাপদ দূরত্ব থেকে দেখছেন (ন্যূনতম 25 সেমি) | | | | | | | |
| 1. লেজারের ব্যবহার- | ☐ কখনই না  ☐ অনিয়মিত  ☐ নিয়মিত | | | | | | | |
| 1. ডেন্টাল প্র্যাক্টিসে ব্যবহৃত চেয়ারের মডেল: |  | | | | | | | |
| 1. কর্মক্ষেত্রে কুলিং সিস্টেমের সুবিধা | ☐ হ্যাঁ ☐ না | | | | | | | |
| 1. দাঁতের অনুশীলনের সময় ব্যবহৃত চেয়ারের মডেল: | ­­­­­­­­­  ____________________________ | | | | | | | |
| **রোগ সংক্রান্ত অন্যান্য তথ্য** | | | | | | | | |
| 1. উচ্চতা (ফুট এবং ইঞ্চি) |  | | | 1. ওজন (কেজিতে) | | |  | |
| 1. আপনি কি লেন্স/চশমা ব্যবহার করেন? | ☐ লেন্স  ☐ চশমা  ☐ লেন্স ও চশমা দুটোই  ☐ কোনটি না | | | | | | | |
| 1. আপনি লেন্স/চশমা ব্যবহার করলে কতবছর ধরে করেন? (শুধুমাত্র সংখ্যা লিখবেন) | | | | | | | | |
| 1. চোখে আঘাতের হিস্ট্রি | ☐ কখনই না  ☐ হ্যাঁ (ডেন্টাল প্র্যাক্টিসের সময়)  ☐ হ্যাঁ (ডেন্টাল প্র্যাক্টিস ব্যতীত) | | | | | | | |
| 1. সর্বশেষ আই কেয়ার ভিসিটের কারণ- | ☐ রুটিন চেক  ☐ পড়তে অসুবিধা  ☐ চোখের লাল হওয়া  ☐ ব্যথা  ☐ চুলকানি  ☐ ডাবল ভিশন  ☐ অন্যান্য | | | | | | | |
| 1. বর্তমানে বিদ্যমান কোনো রোগ | **☐**মাথাব্যথা বা মাইগ্রেন**☐** ছানি**☐** গ্লুকোমা **☐**বারবার কনজেক্টিভাইটিস  **☐**শর্ট সাইটেডনেস**☐**লং সাইটেডনেস**☐** এস্টিগমাটিসম **☐** ডায়াবেটিস মেলিটাস  **☐** উচ্চ রক্তচাপ **☐** অন্যান্য | | | | | | | |
| 1. কোনো ওষুধ নিয়মিত গ্রহণ করে থাকলে তা উল্লেখ করুন |  | | | | | | | |

| **চোখে সমস্যা** **সর্ম্পকিত প্রশ্নাবলী:** | | | |
| --- | --- | --- | --- |
| 1. **চোখে সমস্যা (ফ্রিকোয়েন্সি)** | **কখনই না** | **মাঝে মাঝে** | **প্রায়ই বা সবসময়** |
| জ্বালাপোড়া |  |  |  |
| চুলকানি |  |  |  |
| চোখে ফরেন বডি অনুভূত হয় |  |  |  |
| খোঁচা খোঁচা অনুভূত হয় |  |  |  |
| অত্যধিক পলক |  |  |  |
| লালভাব |  |  |  |
| ব্যাথা |  |  |  |
| ভারী চোখের পাতা |  |  |  |
| শুষ্কতা |  |  |  |
| ঝাপসা দৃষ্টি |  |  |  |
| ডাবল ভিশন |  |  |  |
| কাছাকাছি দৃষ্টির জন্য ফোকাস করতে অসুবিধা |  |  |  |
| আলোর প্রতি সংবেদনশীলতা বৃদ্ধি |  |  |  |
| বস্তুর চারপাশে রঙিন হ্যালো |  |  |  |
| বোধ হয় যে দৃষ্টিশক্তি খারাপ হচ্ছে |  |  |  |
